# Reanalysis of Proteomics Results Fails To Detect MazF-Mediated Stress Proteins

**DOI:** 10.1128/mBio.00949-19

**Published:** 2019-06-11

**Authors:** Niilo Kaldalu, Ülo Maiväli, Vasili Hauryliuk, Tanel Tenson

**Affiliations:** a Institute of Technology, University of Tartu, Tartu, Estonia; b Department of Molecular Biology, Umeå University, Umeå, Sweden; c Laboratory for Molecular Infection Medicine Sweden (MIMS), Umeå University, Umeå, Sweden; Harvard University

**Keywords:** endonuclease, proteomics, statistics, toxin/antitoxin systems

## LETTER

In a recent paper, Nigam and colleagues analyzed the stress-related effects of the endoribonuclease toxin MazF on the Escherichia coli proteome (1). The authors from the lab of Hanna Engelberg-Kulka—the discoverer of the *mazEF* toxin-antitoxin system (2)—claim that MazF creates a unique stress-induced translation machinery (STM). The STM hypothesis states that the toxin cleaves selected mRNAs within 5′-leader sequences to produce a pool of leaderless transcripts that are, in turn, translated by special stress ribosomes (3, 4). The latter are formed when the toxin cleaves off an anti-Shine-Dalgarno sequence-containing fragment from the 3′ end of 16S rRNA in mature ribosomes (3). Thus, MazF is postulated to reshape translation in stressed E. coli similarly to how the σ^S^ factor reshapes transcription.

Independent studies failed to support these findings. Transcriptome-wide mapping of the cleavage sites indicated that MazF cleaves most transcripts within their coding regions and produces very few full-length, leaderless mRNAs (5, 6). Contradicting the STM model, MazF does not cleave rRNA in mature, fully assembled ribosomes but instead targets rRNA precursors (5, 7). Finally, stable isotope labeling by amino acids in cell culture (SILAC)-based proteomics revealed that MazF generally inhibits protein synthesis and no proteins are selectively synthesized in response to the toxin (6). This result is at odds with the paper of Nigam and coworkers (1), who also used SILAC proteomics and report a group of 42 MazF-mediated, stress-induced E. coli proteins. Here we reanalyze their data and highlight several technical issues.

**FIG 1:**
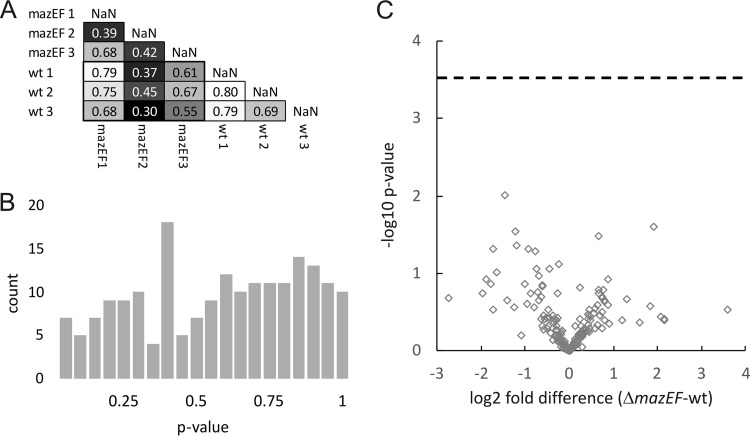
Statistically significant, differentially abundant proteins were not detected upon reanalysis of the proteomics data of Nigam and colleagues (1). H/L ratios of the 192 proteins, which were measured in all three replicate experiments in both E. coli MC4100 *relA*^+^ and Δ*mazEF relA*^+^ strains, were taken from Table S1 in reference 1 and analyzed using the Perseus computational platform (11). (A) *R*^2^ coefficients of determination for individual experiments. (B) Histogram of the Student’s *t* test *P* values. (C) Volcano plot showing differences between the median H/L ratios of individual proteins and their statistical significance. The horizontal dotted line denotes the Bonferroni-corrected (*P* = 0.0003) significance level.

The setup of the proteomics experiment and the lack of statistical analysis make it impossible to determine whether the reported differences in proteomes were caused by MazF or random fluctuations. The authors aimed to test which proteins are synthesized in the Δ*mazEF* mutant and its wild-type (wt) parent strain upon treatment with the quinolone antibiotic nalidixic acid (NA). To do that, Nigam et al. (1) grew bacteria in the light medium, added NA to the culture, and after 10 min, added heavy lysine and arginine in order to label the new proteins. The relative amounts of newly synthetized proteins were estimated based on heavy and light isotope ratio (H/L ratio) after an additional 5-min incubation. The experiment was repeated three times. The short length of pulse labeling resulted in low H/L values, which could, possibly, account for the high variability of results (see below). While the authors state that they “checked several time points and deduced that 5 min is the optimal time point to figure out which are the differential new proteins,” they do not present the relevant supporting data. The authors state that the differences between the wt and Δ*mazEF* proteomes are specifically induced by stress but do not provide an essential control, i.e., proteomic analysis of these strains without NA treatment. Nigam and colleagues admit the lack of statistical analysis and, instead, chose all the proteins “that were induced more in the WT than in the *mazEF* mutants in all the repeats” as differentially expressed. They further state that “as the purpose of the study was to identify the new proteins rather than to calculate the turnover of the proteins, no complex statistical test was used and no logarithmic transformation was done”. We statistically reanalyzed the data to control for the false-positive rate of assignment into the group of differentially expressed proteins. We found similar levels of covariation between the intrastrain replicate experiments and interstrain comparisons (Fig. 1A), while no spike of small *P* values appeared on the *P* value histogram obtained from Student’s *t* test of log_2_-transformed data (Fig. 1B). This result is consistent with the null hypothesis of no differentially expressed proteins, which results in a flat distribution of *P* values. A volcano plot demonstrates an almost equal number of overexpressed and less-expressed heavy proteins in the Δ*mazEF* mutant strain compared to the wild type, while no *P* values surpass the Bonferroni-corrected significance level (Fig. 1C). We also could not detect any differentially expressed proteins at a false-discovery rate (FDR) of 0.1 using a less conservative Benjamini-Hochberg method. The lowest q-value for a particular protein was 0.88, which means that we can accept this protein as differentially expressed only at a 0.88 false-discovery rate level.

The technical issues compromising the SILAC analysis are further confounded by the lack of experimental validation of MazF activation and cutting of the mRNA leader sequences at the listed sites (see Table 1 in reference 1) upon NA treatment. NA targets type II topoisomerases but does not inhibit RNA or protein synthesis and is not expected to stop production of the MazE antitoxin to activate the toxin. The authors refer to a paper that reports NA-triggered, MazF-mediated programmed cell death (PCD) but does not present evidence of RNA fragmentation (8). Other researchers could not reproduce the *mazEF*-dependent PCD (9) and have found that the E. coli MC4100 *relA*^+^ and Δ*mazEF relA*^+^ strains used by Nigam et al. harbor a frameshift mutation in *relA* and are phenotypically *relA* deficient (relaxed, *relA* mutant [9, 10]). Inactivation of the *relA-*mediated stringent response, a central mechanism of stress adaptation, further complicates interpretation of the results.

Finally, we note the absence of citations to papers critical of the STM hypothesis (5–7).
